# Photoacoustics resolves species-specific differences in hemoglobin concentration and oxygenation

**DOI:** 10.1117/1.JBO.25.9.095002

**Published:** 2020-09-04

**Authors:** Lina Hacker, Joanna Brunker, Ewan St. John Smith, Isabel Quiros-Gonzalez, Sarah E. Bohndiek

**Affiliations:** a University of Cambridge, Department of Physics, Cambridge, United Kingdom; b University of Cambridge, Cancer Research UK Cambridge Institute, Cambridge, United Kingdom; c University of Cambridge, Department of Pharmacology, Cambridge, United Kingdom

**Keywords:** photoacoustic imaging, hemoglobin, phantom, oxygenation

## Abstract

**Significance:** Photoacoustic imaging (PAI) enables the detection of blood hemoglobin (HB) concentration and oxygenation (${\mathrm{sO}}_{2}$) with high contrast and resolution. Despite the heavy use of photoacoustically determined total hemoglobin (THb) and oxygenation (${\mathrm{sO}}_{2}$) biomarkers in PAI research, their relationship with underlying biochemical blood parameters and the impact of intra- and interspecies genetic variability have yet to be established.

**Aim:** To explore the relationship between THb and ${\mathrm{sO}}_{2}$ photoacoustic biomarkers and the underlying biochemical blood parameters in a species-specific manner.

**Approach:** Experiments were performed on blood *in vitro* using tissue-mimicking agar phantoms. Blood was extracted from mouse, rat, human, and naked mole-rat (*Heterocephalus glaber*), anticoagulated in ethylenediaminetetraacetic acid, and measured within 48 h. THb and ${\mathrm{sO}}_{2}$ were measured using a commercial photoacoustic tomography system (InVision 128, iThera Medical GmBH). Biochemical blood parameters such as HB concentration (g/dL), hematocrit (HCT, %), and red blood cell (RBC) count (${\mu L}^{-1}$) were assessed using a hematology analyzer (Mythic 18 Vet, Woodley Equipment).

**Results:** A significant correlation was observed between THb and biochemical HB, HCT, and RBC in mouse and rat blood. Moreover, PAI accurately recapitulated interspecies variations in HB and HCT between mouse and rat blood and resolved differences in the oxygen dissociation curves measured using ${\mathrm{sO}}_{2}$ between human, mouse, and rat. With these validation data in hand, we applied PAI to studies of blood obtained from naked mole-rats and could confirm the high oxygen affinity of this species in comparison to other rodents of similar size.

**Conclusions:** Our results demonstrate the high sensitivity of photoacoustically determined hemoglobin biomarkers toward species-specific variations *in vitro*.

## 1 Introduction

Photoacoustic imaging (PAI) is an emerging modality able to reveal high image contrast, arising from optical absorption in tissue at high spatial resolution, afforded by ultrasound detection. PAI is based on the photoacoustic principle,^1^ whereby pulsed light is absorbed by chromophores resulting in the generation of pressure waves that can be detected by ultrasound transducers at the tissue surface. Applying PAI at multiple wavelengths enables noninvasive, label-free detection of total hemoglobin concentration (THb) and oxygenation (${\mathrm{sO}}_{2}$) based on the different optical absorption spectra of deoxygenated (HbR) and oxygenated hemoglobin (${\mathrm{HbO}}_{2}$).^2^ The relative concentrations of these respective chromophores can then be calculated by spectroscopic inversion.^3^ PAI measures of THb ($\mathrm{HbR}+{\mathrm{HbO}}_{2}$) and ${\mathrm{sO}}_{2}$ (${\mathrm{HbO}}_{2}/\mathrm{THb}$) have been widely exploited to characterize tissues in the context of different pathologies, for example in breast cancer,^4^^–^^6^ melanomas,^7^^,^^8^ prostate cancer,^9^^–^^11^ nodal lesions of the head and neck,^12^ vascular diseases,^13^^–^^15^ and colitis.^16^ Particularly in cancer biology, THb and ${\mathrm{sO}}_{2}$ have proved to be of high value by allowing the detection of two cancer hallmarks: angiogenesis and hypoxia.^17^

Despite their extensive use in PAI, the relationship between photoacoustically determined THb and ${\mathrm{sO}}_{2}$ biomarkers and the underlying physiological variations in biochemical blood parameters, such as hemoglobin (HB) concentration (g/dL), hematocrit (HCT, %), and red blood cell (RBC) count (${\mu L}^{-1}$), has yet to be established. HB is an iron-containing hem tetramer composed of two α- and two β-monomers and responsible for oxygen transport in all vertebrates,^18^ yet HB-related blood parameters differ within and between species and human race,^19^^,^^20^ with age and sex^21^^–^^23^ of the individual, and can change under pathophysiological conditions or pharmaceutical treatment.^24^ Moreover, genetic modifications of the HB protein sequence resulting from either accumulated evolutionary changes or spontaneous mutations can alter the oxygen binding capabilities of the HB molecule.^25^^–^^27^ Such differences could lead to substantial variations in PAI biomarkers or, for example, signal dynamics during more complex PAI protocols such as a gas challenge,^10^ which could lead to misinterpretation of PAI data.

Here, we investigate the relationship of photoacoustically determined THb and ${\mathrm{sO}}_{2}$ biomarkers with biochemical blood parameters taken from mice, rats, and humans in a controlled tissue-mimicking phantom system. Having examined the intraspecies homogeneity of the globin genes, we first elucidate intra- and interspecies differences between photoacoustic THb and static biochemical blood parameters in mouse and rat. We then move on by examining the differences in the dynamics of the oxygen dissociation curves (ODCs), here including human as a further species. With these validation data in hand, we then apply PAI to studies of blood obtained from naked mole-rats (*Heterocephalus glaber)*, a species remarkable for its resistance to oxygen deprivation.^28^ Our results confirm the high sensitivity of photoacoustically determined biomarkers toward species-specific variations *in vitro*, which should be considered in future study designs.

## 2 Materials and Methods

### 2.1 Sequencing

Deoxyribose nucleic acid (DNA) was isolated from liver samples of female C57BL/6J mice (3 to 4 months, n=3) and Wistar rats (6 to 9 months, n=3) (Charles River) using the Qiagen DNeasy Blood/Tissue kit (Cat. no. 69504) following the manufacturer’s instruction. Primers (displayed in Table 1) for both the forward and reverse strand of each gene were designed using Primer 3 software (version 4.0.0).^29^ Polymerase chain reaction (PCR) amplification was performed using the Q5^®^ Hot Start High-Fidelity DNA Polymerase (New England BioLabs Inc.) and the corresponding protocol^30^ with an annealing temperature of 66°C. Products were purified and sequenced using Sanger sequencing (SourceBioscience). Sequences were aligned using Clustal Omega.^31^ The identity match between the sequences was calculated.

**Table 1:** Confirmed intraspecies gene sequence identity of HB α- and β-genes in female mouse (n=3) and rat (n=3).

Species	Gene	Primer sequence		Intraspecies gene identity (%)
Mouse	HBA-A1	Exon 1+2	F: GGGCAACTGATAAGGATTCCC	100
			R: GACCACTATGTTCCCTGCCT	
		Exon 3	F: TGTCCACTTTGTCTCCGCA	100
			R: ACATGACACCTTTGCAGACG	
	HBA-A2	Exon 1+2	F: CTACTTGCTGCAGGTCCAA	100
			R: CCAGGTCCCAGCGCATAC	
		Exon 3	F: TGTCCACTTTGTCTCCGCA	100
			R: AGAAGCGTCCCCACACTAAA	
	HBB-t	Exon 1+2	F: TCATCTCTGAAGCCTCACCC	100
			R: ATAGCCAGGGGAAGGAAACC	
Rat	HBA-A1	Exon 1+2	F: GAAACTTGCTGCAGGGTCAA	100
			R: GCCAGGTCTGAGCTCACA	
	HBA-A2	Exon 1+2	F: CAATGACAGCTGCTCCAAGG	100
			R: CAAGGGATCTCTGGAGGACC	
	HBA-A3	Exon 1+2	F: GCTGCAGGGCCAATACATTC	100
			R: GCCAGGTCTGAGCTCACA	
	HBB	Exon 1+2	F: ATTGGCCAATCTGCTCACAC	100
			R: GAAAGCCACAGGAAGGACAC	

### 2.2 Blood Samples

Whole blood and tissue samples (liver) for DNA extraction originating from female C57BL/6J mice (3 to 4 months) and Wistar rats (6 to 9 months) were ordered from Charles River Laboratories. For comparison of the blood parameters in Secs. 1–3, the same sex was chosen as HB parameters have been shown to be sensitive to the sex of individuals.^22^ For the blood oxygenation experiments in the remaining Secs. 4 and 5, blood from mixed sex was used to achieve more generalizable results. Human blood samples from healthy donors were collected under the research ethics approval of the Royal Papworth Hospital tissue bank (project number T02196) in Cambridge. Blood from naked-mole rats (15 to 23 months, all male) was collected as a postmortem nonregulated procedure following decapitation of the animal for another scientific purpose. All blood samples were anticoagulated in ethylenediaminetetraacetic acid, directly stored at 4°C, and processed within 48 h.

### 2.3 Blood Analysis

Blood parameters were assessed using an impedance-based hematology analyzer (Mythic 18, Woodley Veterinary Diagnostics^32^). The parameters obtained included: absolute and relative number of lymphocytes, monocytes, and granulocytes; absolute number of RBC (${\mu L}^{-1}$) and white blood cells (WBC, ${\mu L}^{-1}$); HB concentration (g/dL); mean corpuscular hemoglobin (MCH, pg); mean corpuscular volume (MCV, ${\mu m}^{3}$); mean corpuscular hemoglobin concentration (MCHC, g/dL); and HCT (%). MCV, MCHC, and MCH are commonly defined as: MCV = [HCT]/RBC, MCHC = [HB]/[HCT], and MCH = [HB]/RBC, where square brackets denote concentrations.

### 2.4 Absorption Spectra Measurement

The absorption profile of HB was independently verified. Whole blood was lysed in distilled water (1:1) and the HB extracted in order to avoid artifacts caused by light scattering of the erythrocytes. For HB extraction,^33^ blood was centrifuged three times at 3000 rpm for 3 min and washed with phosphate buffered saline (PBS) to remove the plasma. Afterward, 1 unit volume of blood was thoroughly mixed with 1 unit volume of deionized water and 0.4 unit volume of toluene. The mixture was stored at 4°C for at least 24 h to ensure complete hemolysis. The solution was then centrifuged at 13,000 rpm for 10 min. The lowest layer containing the HB was extracted by syringe and filtered through a syringe filter (Millex, Millipore) with a pore size of 0.22  μm to remove the cell debris and large particles. The absorption spectrum of extracted HB was recorded in the range of 600 to 900 nm using a microplate reader (Clariostar, BMG Labtech). The absorption spectra were normalized to the area under the curve to account for the different HB levels of rat and mouse blood.

### 2.5 Phantom Preparation and Photoacoustic Imaging

Agar phantoms were produced according to the protocol by Joseph et al.^36^ Briefly, liquid 1.5% w/v agar (Fluka 05039) was mixed with 2.1% v/v prewarmed intralipid (Sigma-Aldrich I141) to provide a reduced scattering coefficient of $5\;{\mathrm{cm}}^{-1}$. Nigrosin dye (Sigma-Aldrich 198285) was added to provide an absorption coefficient of $0.05\;{\mathrm{cm}}^{-1}$ (at 564 nm, peak of the nigrosin spectrum). The solution was poured into a 20-mL (2-cm diameter) syringe with the injection end removed and with polyvinyl chloride tubing (inner diameter: 1.5 mm, outer diameter: 2.1 mm; VWR 228-3857) inserted along the central axis. After the agar was set, the phantom was removed from the syringe ready for imaging.

PAI experiments were performed using a commercial small animal imaging system (MSOT inVision 256-TF; iThera Medical GmbH). The system has been described in detail elsewhere.^37^^,^^38^ Briefly, a tunable (660 to 1300 nm) optical parametric oscillator (OPO), pumped by a nanosecond (ns) pulsed Nd:YAG laser, with 10-Hz repetition rate and up to 7-ns pulse duration provides excitation pulses. Light is delivered to the sample through a custom optical fiber assembly, which creates a diffuse ring of uniform illumination over the imaging plane. The sample is coupled to the transducers using a water bath, filled with degassed, and deionized water. For ultrasound detection, 256 toroidally focused ultrasound transducers covering an angle of 270 deg are used (center frequency of 5 MHz, 60% bandwidth) allowing tomographic reconstruction. A minimum of four images were taken along the length of the phantom at steps of 0.5 mm using seven wavelengths (700, 730, 760, 800, 850, 900, and 1040 nm) with an average of 10 pulses per wavelength.

For the measurements of the ODC, a flow phantom set up was used, which has also been described in detail elsewhere.^39^ For each measurement, 5 mL of pooled blood from the respective species was first oxygenated by the addition of 0.2% v/v hydrogen peroxide [$H_{2}O_{2}$ 30% (w/w) in deionized water, Sigma-Aldrich 7722-84-1]. The oxygenated blood was filled into the circuit, carefully avoiding the introduction of air bubbles. During the course of the experiment, a syringe driver (Harvard, MKCB2159V) was used to deoxygenate the blood with 0.03% w/v sodium hydrosulfite (ACROS Organics 7775-14-6) in PBS) at a constant flow rate of 10  μL/min. The experiment was performed at room temperature and a peristaltic pump (Fisher Scientific CTP100) was used for blood circulation. Oxygen fluorescence quenching needle probes (Oxford Optronix, NX-BF/O/E) were placed before and after the tissue-mimicking phantom, which recorded the temperature and partial pressure of oxygen (${\mathrm{pO}}_{2}$, mmHg) in real time. The data were downloaded via an Arduino UNO and read in MATLAB^®^. Using the same commercial PAI system as above, images were acquired at a single position (no pulse-to-pulse averaging) for 17 wavelengths (660, 664, 680, 684, 694, 700, 708, 715, 730, 735, 760, 770, 775, 779, 800, 850, and 950 nm). Absorption spectra were measured using a light source (Avantes Avalight-HAL-S-Mini) and spectrometer (AvaSpec-ULS2048-USB2-VA-50). Absorption spectra were recorded continuously via AvaSoft software as the fluid passed through a flow cell (Hellma Analytics, 170700-0.5-40) as it has been shown that directly measured absorption spectra provide the most accurate endmembers for spectral unmixing.^39^ Species-specific absorption spectra at the point of complete oxygenation and deoxygenation were extracted and used for spectral unmixing of the data recorded for the respective species. Between each measurement run, the tubing containing the blood was cleaned with PBS.

For the experiments involving naked mole-rat blood, a simpler set up was used as only a limited amount of naked mole-rat blood could be obtained. Blood samples (100  μL) were inserted into a straw within a phantom and 10  μL $H_{2}O_{2}$ (0.03% in PBS) was injected using a syringe. The oxygenation of the blood was directly measured after injection using MSOT. Thirty images were taken of a chosen slice using 7 wavelengths (700, 730, 760, 800, 850, 900, and 1040 nm), with an average of 10 pulses per wavelength. Each image took 7 s to acquire.

Measurements were conducted at 37°C. It should be noted here that naked-mole rats are considered poikilothermic and usually have a physiological body temperature of around 30°C to 32°C.^40^ Examining the oxygen affinity at higher temperature decreases the oxygen affinity of the blood.^41^ However, it has been shown that at 37°C significant differences between the oxygen affinity of naked mole-rat and mouse are still present,^42^ supporting our experimental approach.

### 2.6 Image and Statistical Analysis

PAI data were analyzed using the ViewMSOT software (v3.6.0.119; iThera Medical GmbH). Model-based image reconstruction and linear multispectral processing were applied to extract the relative signal contributions of ${\mathrm{HbO}}_{2}$ and HbR. The same position within the phantom was used to determine the average intensities for ${\mathrm{HbO}}_{2}$ and HbR. Regions of interest (ROIs) were manually drawn around the circular cross section of the tube inserted in the phantom. ${\mathrm{THb}}^{\text{MSOT}}$ was calculated as the sum of ${\mathrm{HbO}}_{2}$ and HbR. ${\mathrm{sO}}_{2}^{\mathrm{MSOT}}$ was calculated as the ratio of ${\mathrm{HbO}}_{2}$ to ${\mathrm{THb}}^{\text{MSOT}}$ signal. The expressions ${\mathrm{THb}}^{\text{MSOT}}$ and ${\mathrm{sO}}_{2}^{\mathrm{MSOT}}$ are used to emphasize that the photoacoustically determined THb and ${\mathrm{sO}}_{2}$ biomarkers are not exactly equal to the underlying parameters, since to be able to accurately resolve absolute values, knowledge of the light fluence distribution, system response, and Grüneisen parameter is required.^43^ For dynamic experiments, a trendline was fitted on the increasing ${\mathrm{sO}}_{2}$ values and the maximum value reached during the experiment and gradient of the trendline were extracted, to be compared between the species.

Raw data extracted from the ROIs were analyzed using Python (version 2.7) and MATLAB. Statistical analysis was performed using Prism (GraphPad). All data are shown as mean±standard error of the mean (SEM) unless otherwise stated. Unpaired two-tailed t-tests were performed to calculate the statistics. Pearson’s rank test was performed to assess correlations between biochemical blood parameters and ${\mathrm{THb}}^{\text{MSOT}}$. Significance is assigned for p-values <0.05.

## 3 Results

### 3.1 Gene sequence Analysis Confirms Intra- and Interspecies Homogeneity of Hemoglobin Genes in Mouse and Rat

We first assessed the intraspecies genetic correspondence of the two α- and two β-globin chains of the HB tetramer in rat and mouse, as alterations in HB globin genes can lead to changes in the protein structure that might affect the PAI signal. We found that an intraspecies homogeneity of 100% could be determined (n=3, Table 1). These results indicate that intraspecies signal variations in the following experiment are unlikely to be caused by genetic missense mutations, but rather by other physiological or technical sources.

### 3.2 PAI THb Correlates Directly with Biochemical HB Values Obtained Using a Hematology Analyzer and Detects Intraspecies Variations

**Fig. 1:**
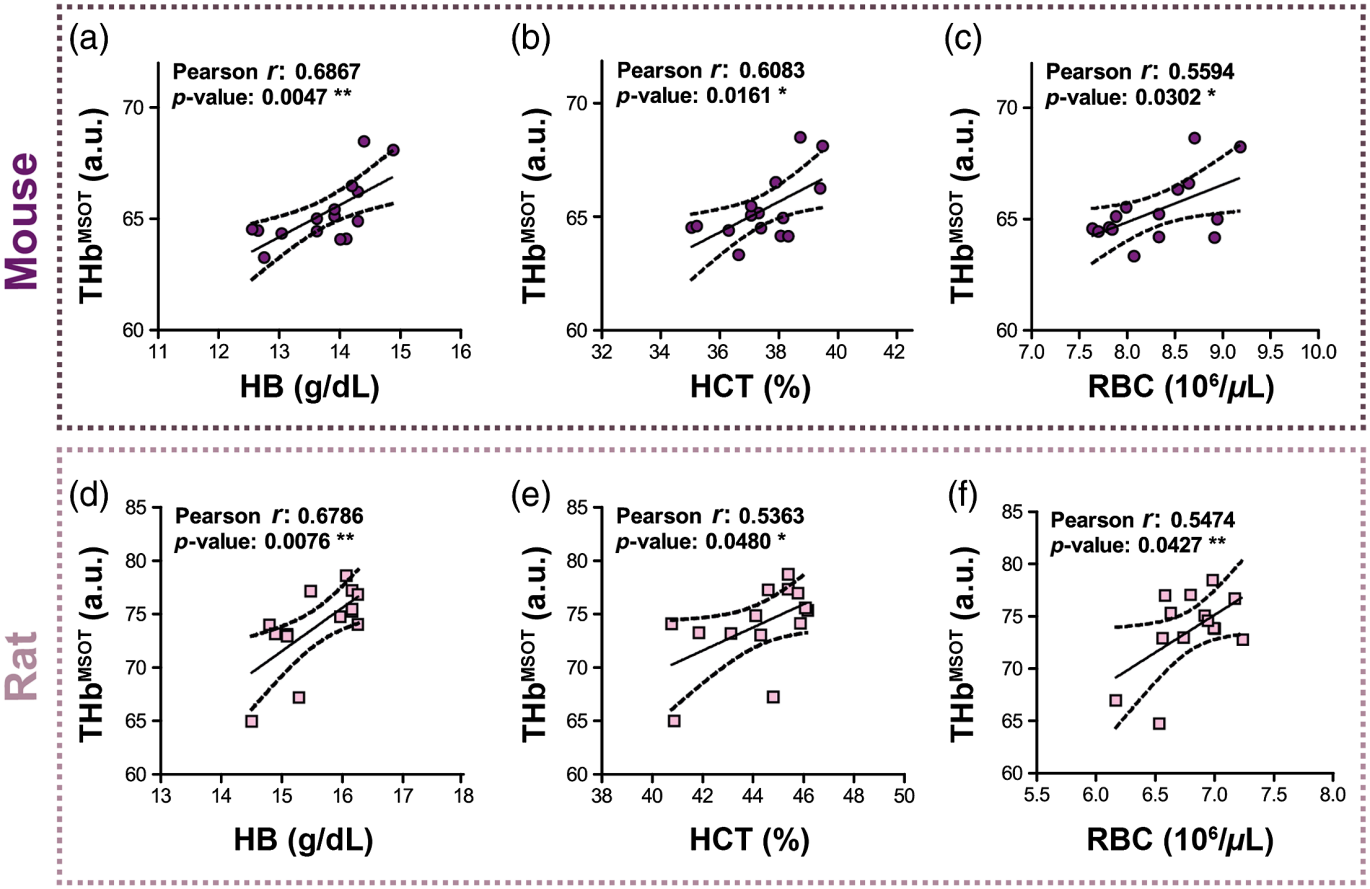
${\mathrm{THb}}^{\text{MSOT}}$ biomarker measured in tissue-mimicking phantoms corresponds to biochemical HB, HCT, and RBC levels in mouse and rat. ${\mathrm{THb}}^{\text{MSOT}}$ extracted from PAI data obtained from tissue-mimicking phantoms containing blood from female mice [n=15, purple circles, (a)–(c)] and rats [n=14, pink squares, (d)–(f)] correlated significantly with biochemical (a), (d) HB; (b), (e) HCT; and (c), (f) RBC count concentration in both species. * p<0.05, ** p<0.01 by Pearson correlation.

The relationship between the ${\mathrm{THb}}^{\text{MSOT}}$ biomarker and biochemical blood parameters was examined. ${\mathrm{THb}}^{\text{MSOT}}$ values from photoacoustic images taken from our static blood phantoms correlated with biochemical HB [Fig. 1(a)], HCT [Fig. 1(b)], and RBC [Fig. 1(c)] in the same mouse blood sample (HB: Pearson r=0.6867, p=0.0047; HCT: Pearson r=0.6083, p=0.0161; RBC: Pearson r=0.5594, p=0.0302). To determine whether these observations are species-independent, the correlation between ${\mathrm{THb}}^{\text{MSOT}}$ and biochemical blood parameters was also studied in rat. Again, a significant correlation was found between ${\mathrm{THb}}^{\text{MSOT}}$ and biochemical HB [Fig. 1(d)], HCT [Fig. 1(e)], and RBC [Fig. 1(f)] in rat blood (HB: Pearson r=0.6786, p=0.0076; HCT: Pearson r=0.5363, p=0.0480; and RBC: Pearson r=0.5474, p=0.0427) suggesting that PAI is directly sensitive to intraspecies variations in HB, HCT, and RBC parameters.

### 3.3 PAI THb Resolves Interspecies Differences in HB and HCT* in vitro*

**Fig. 2:**
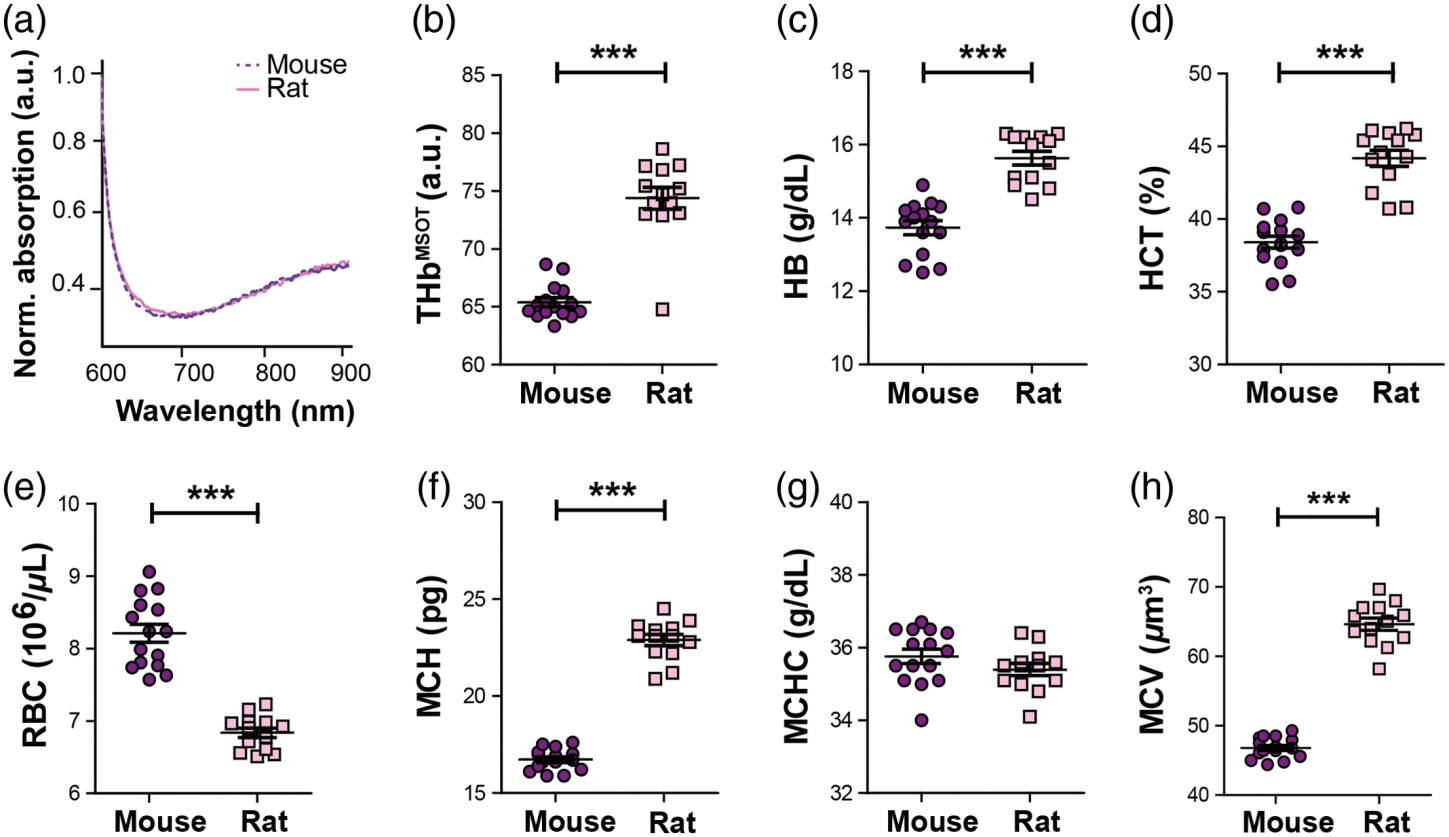
PAI resolves interspecies HB differences in rat and mouse. (a) Absorption spectra of HB extracted from lysed whole blood of mouse (purple) and rat (pink). (b) Interspecies differences in ${\mathrm{THb}}^{\text{MSOT}}$ obtained from PAI measurements of female mouse (n=15) and rat (n=14) blood show a significantly higher value in rat blood, which was underscored by differences in biochemical HB (c) and HCT (d). Notably, the RBC value (e) was found to be lower in the rat, but with higher mean corpuscular hemoglobin (MCH) per red blood cell (f). While the mean corpuscular hemoglobin concentration (MCHC) was comparable between the species (g), the mean corpuscular volume MCV (h) was higher in the rat. Data are represented as mean±SEM, significance *** p<0.0001 by unpaired t-test.

After assessing the impact of intraspecies variation in biochemical blood parameters on MSOT signal, the effect of interspecies differences was analyzed. In order to exclude major differences in the protein structure and conformation yielding differences in the absorption spectra for the HB, we first confirmed that no interspecies difference in the absorption spectra of HB extracted from mouse and rat blood was detected. Minor differences in total light absorbance of the samples were observed, which could be explained by different HB levels of rat and mouse blood (Table 2); thus, absorption spectra were normalized to the area under the curve for further comparison. In line with the literature,^44^^,^^45^ the experimentally determined spectra of mouse and rat blood demonstrated a very good agreement [Fig. 2(a)].

**Table 2:** Comparison of experimental and literature HB and HCT values of mouse (n=15) and rat (n=14).

Parameter	Mouse (BALB/c)	Rat (Wistar)
HB (exp.) (g/dL) (mean±std)	14.73±2.09	15.06±2.35
HB (lit.) (g/dL)	11 to 16	14.4 to 18.0
HCT (exp.) (%) (mean±std)	41.83±6.38	45.50±6.13
HCT (lit.) (%)	37 to 52	36 to 48
Reference	Santos et al. (2016)^34^	Charles River Laboratories, 1998^35^

Next, ${\mathrm{THb}}^{\text{MSOT}}$ was compared to the biochemical blood parameters of the different species. A significantly higher ${\mathrm{THb}}^{\text{MSOT}}$ level was observed for the rat [p<0.0001, Fig. 2(b)]. Corresponding to this observation, significantly higher HB [Fig. 2(c)] and HCT [Fig. 2(d)] values could be observed in this species (p<0.0001). Interestingly, the rat was characterized by significantly lower RBC levels [Fig. 2(e)]. We compared the MCH levels between the two species to establish whether this observation was associated with differences in the average HB amount per RBC. In correspondence to the HB and HCT values, significant higher MCH levels were found in the rat [Fig. 2(f), p<0.0001]. A more detailed analysis revealed that the higher MCH values in the rat do not originate from a higher MCHC value [Fig. 2(g)], but rather from a larger MCV of the RBC [p<0.0001, Fig. 2(h)]. These results suggest that PAI correctly resolves interspecies differences in HB and HCT *in vitro*, but can only be used to quantitatively compare interspecies RBC values when MCH values are within the same range.

### 3.4 PAI Resolves Interspecies Differences in Oxygenation Dynamics

**Fig. 3:**
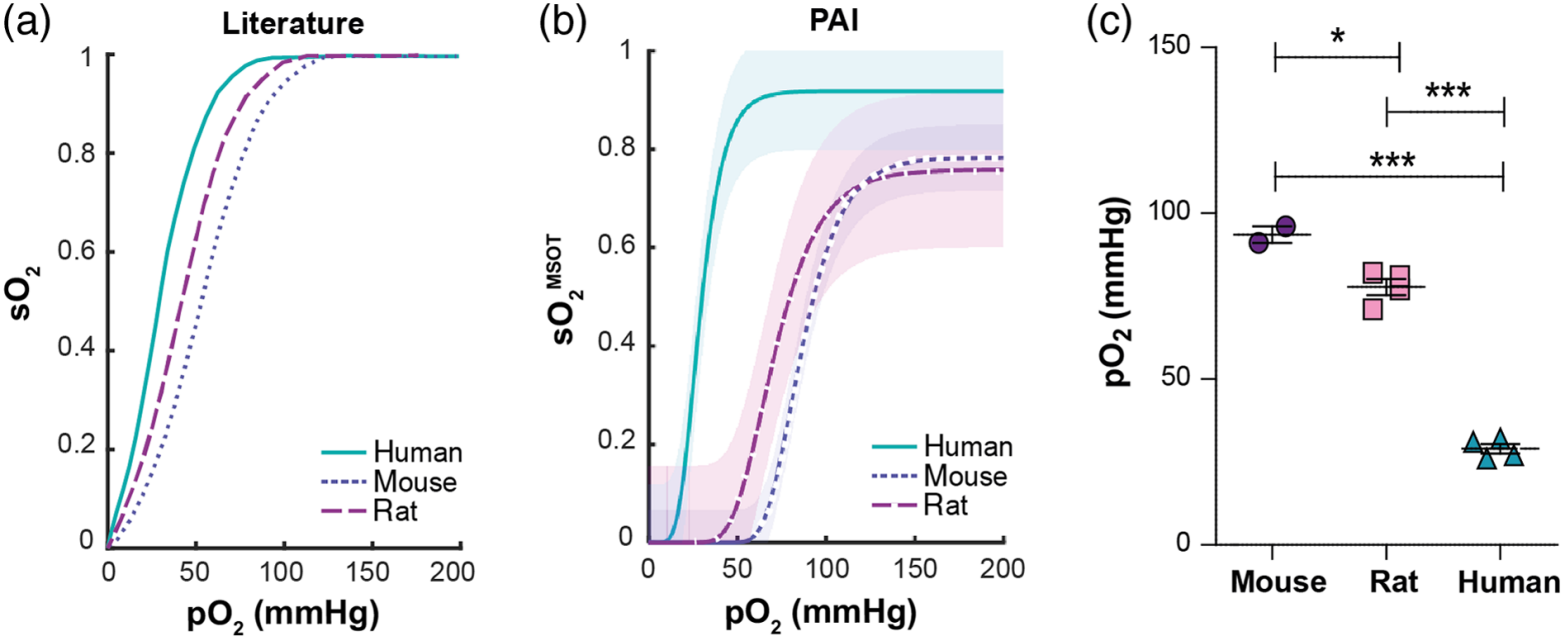
PAI resolves interspecies differences in ODCs between mouse, rat, and human. Oxygenated mouse (n=2), rat (n=4), and human (n=5) blood samples were deoxygenated in a dynamic flow phantom circuit at room temperature and changes in absorption spectrum, ${\mathrm{pO}}_{2}$, and PAI signals were recorded. (a) Literature values for ODCs of the respective species under standard conditions.^26^ (b) ${\mathrm{sO}}_{2}^{\mathrm{MSOT}}$ was calculated from PAI images using the absorption spectra measured within the flow phantom circuit for spectral unmixing. The resulting values were plotted against the measured ${\mathrm{pO}}_{2}$ of the blood within the circuit at the same time point in order to produce an ODC. (c) Extracted ${p50}^{\mathrm{MSOT}}$ values denoting the ${\mathrm{pO}}_{2}$ at 50% ${\mathrm{sO}}_{2}^{\mathrm{MSOT}}$ are shown. It should be noted that the ODC influencing factors 2,3-DPG concentration, acid–base balance, and amount of dyshemoglobins were not standardized, but rather reflect the physiological values found in the respective species.

We next examined whether PAI has sufficient sensitivity to capture interspecies differences in oxygenation dynamics in mice, rat, and human blood based on the known differences in ODCs in the literature between these species [Fig. 3(a)].^26^ Under standard conditions [pH=7.4, ${\mathrm{pCO}}_{2}=40\;\mathrm{mmHg}$ (5.3 kPa), temperature = 37°C, carboxyhemoglobin <2%], human HB is known to have the highest oxygen affinity with an ODC shifted farthest to the left (${p50}_{\mathrm{std}}=26\;\mathrm{mmHg}$), followed by rat (${p50}_{\mathrm{std}}=32\;\mathrm{mmHg}$), and then mouse HB (${p50}_{\mathrm{std}}=48.5\;\mathrm{mmHg}$)^26^ [Fig. 3(a)].

To test whether MSOT is able to qualitatively resolve these differences, fully oxygenated blood was introduced into a dynamic flow phantom system and the blood was gradually deoxygenated while the absorption spectra, ${\mathrm{pO}}_{2}$, temperature, and PAI spectral data were recorded in real time. The species-specific ODCs were compared by plotting ${\mathrm{sO}}_{2}^{\mathrm{MSOT}}$ against ${\mathrm{pO}}_{2}$ and showed broadly similar results as reported in the literature^25^^,^^26^ [Fig. 3(b)]. In a similar pattern, the human ODC was found to be shifted farthest to the left (${p50}_{\mathrm{MSOT}}=29.00\pm 2.94\;\mathrm{mmHg}$), followed by the rat (${p50}_{\mathrm{MSOT}}=77.75\pm 4.99\;\mathrm{mmHg}$), and then the mouse (${p50}_{\mathrm{MSOT}}=93.50\pm 3.54\;\mathrm{mmHg}$) [Fig. 3(c)]. These results indicate that MSOT is capable of qualitatively resolving interspecies oxygenation dynamics *in vitro*.

### 3.5 PAI is Sensitive to the Enhanced Oxygen Dissociation Curve of the Naked Mole-Rat

Having established the capacity for PAI to resolve differences in both HB parameters and oxygenation dynamics in mice and rats, we made a first PAI study of blood from the naked mole-rat. The naked mole-rat is adapted to live in a hypoxic underground environment, which involves having a higher affinity for oxygen in its HB molecules than rodents of similar size.^42^

**Fig. 4:**
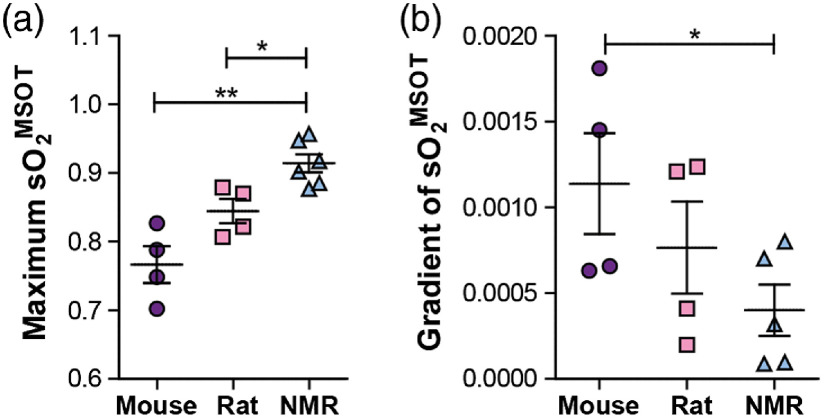
PAI evaluation of oxygenation dynamics is sensitive to the higher oxygen affinity of naked mole-rat blood. Blood samples taken from mouse (n=4), rat (n=4), and naked mole-rat (NMR; n=5) were oxygenated using 10  μL hydrogen peroxide (0.03% in PBS) at 37°C. (a) The maximum ${\mathrm{sO}}_{2}^{\mathrm{MSOT}}$ was highest in the naked mole-rat. (b) Considering the sigmoidal shape of the ODC, this would be expected to lead to the lowest gradient of the oxygenation change, which was confirmed in our experiments.

As only small amounts of naked mole-rat blood could be obtained, a simpler experimental design was used for the studies in which small blood samples were oxygenated with $H_{2}O_{2}$ (0.03% in PBS) and the oxygenation measured using PAI during 4 min. A significantly higher maximum ${\mathrm{sO}}_{2}^{\mathrm{MSOT}}$ after 4 min could be found for the naked mole-rat blood in comparison to the same experiment conducted with mouse and rat blood [Fig. 4(a)]. Some deviation is observed between the maximum ${\mathrm{sO}}_{2}^{\mathrm{MSOT}}$ obtained for the rat in this experiment compared to the earlier findings (Fig. 3), although it is still within the bounds of error. The deviation is likely due to experiments being conducted on different batches of blood provided by an external supplier, which may have experienced different extractions or handling beyond our control. Considering the sigmoidal shape of the ODC, the highest maximum ${\mathrm{sO}}_{2}^{\mathrm{MSOT}}$ observed in the naked mole-rat should be accompanied with the lowest gradient of the oxygenation change, which was indeed found in our experiments [Fig. 4(b)]. The results suggest that PAI is able to resolve the higher oxygen affinity of the naked-mole rat blood^42^ compared to mouse and rat, indicated by the overall higher ${\mathrm{sO}}_{2}$ and lower change of ${\mathrm{sO}}_{2}$ when oxygenating the samples under similar experimental conditions.

## 4 Discussion

PAI holds substantial potential for application in the measurement of THb and ${\mathrm{sO}}_{2}$ biomarkers, however, our understanding of the relationship between these biomarkers and the biochemical parameters of blood including HB concentration, HCT, and ODCs has yet to be fully elucidated. Here, we sought to study the sensitivity of PAI toward physiological variations of HB, including both intra- and interspecies variations.

Our results indicate that PAI determined ${\mathrm{THb}}^{\text{MSOT}}$ and ${\mathrm{sO}}_{2}^{\mathrm{MSOT}}$ can resolve physiological variations in HB and HCT both within and between species. We found significant linear correlations of ${\mathrm{THb}}^{\text{MSOT}}$ with HB, HCT, and RBC count. As HCT and HB linearly depend on each other (roughly HB=HCT/3),^46^ it is unsurprising that both show linear trends. Our findings indicate that when the concentration of corpuscular HB is comparable between subjects, differences in RBC count could, in principle, be determined using PAI, however, this would require a low variance of the corpuscular HB value within the investigated group of subjects. Even within species, significant variations in MCH can occur due to disease-related macro- or microcytic anemia or age,^47^ which should be considered when making any conclusions from PAI signal to RBC number.

We could further show that differences in interspecies oxygenation dynamics based on the measurement of the PAI biomarker ${\mathrm{sO}}_{2}^{\mathrm{MSOT}}$ could be clearly resolved. This is notable given the diversity of species studied. In particular, our studies revealed that ${\mathrm{sO}}_{2}^{\mathrm{MSOT}}$ is sensitive to the differences in ODCs between mouse, rat, and human, as well as naked mole-rat. It has to be noted that our study only aimed for a qualitative comparison of the ODC curves between the species. For a quantitative comparison of ODC characteristics and p50 values, other ODC influencing factors, such as species-specific 2,3-diphosphoglycerate (2,3-DPG) concentration,^48^ pH,^49^ amount of dyshemoglobins,^41^ or the partial pressure of carbon dioxide (${\mathrm{pCO}}_{2}$)^50^ would also need to be taken into consideration.

Our study indicates that the application of PAI to detect functional differences in HB should be considered carefully when comparing data obtained from different species, particularly when using an “oxygen-enhanced” or “gas challenge” imaging protocol.^10^^,^^51^ It also highlights the exciting potential of PAI derived biomarkers to be applied for studies in disease-associated anaemia^52^ or hemoglobinopathies, such as sickle cell anemia.^53^ Patients with hemoglobinopathies where globin proteins are structurally abnormal often show shifts in the ODC,^54^ which may be captured using PAI.

Despite these promising findings, there remain some limitations of the study. We used a standard linear spectral unmixing method for resolving the contributions of HbR and ${\mathrm{HbO}}_{2}$ to our signals, which produced values of ${\mathrm{sO}}_{2}^{\mathrm{MSOT}}$ with limited dynamic range, particularly at the higher end of the ODCs, when compared to ground truth. Employing more advanced multispectral processing techniques should further enhance the accuracy of the photoacoustically determined ${\mathrm{sO}}_{2}$ estimation, however, improved spectral classification methods remain a topic of active research in the field and an optimal solution has yet to be reached.^55^^–^^57^

Further, our *in vitro* phantom experiments provide a well-controlled reference measurement for both the HB absorption spectrum and the partial pressure of oxygen. Although it may be possible in future work to use the knowledge of interspecies variations observed in this study to make a qualitative *in vivo* comparison of the PAI biomarker response, for further validation of our findings, *in vivo* confirmation would be advantageous. Unfortunately, the experimental design would be complex because of the imaging artifacts that arise due to: tissue heterogeneities, which lead to local variations in optical and acoustic properties, and consequently to uncertainty in PAI fluence distributions^58^^,^^59^ motion, such as breathing or heartbeart;^60^ and anatomical positioning within a nonrigid animal holder.^36^ Furthermore, factors influencing blood extraction, such as blood clotting, hemolysis, dilution of blood with interstitial fluid, or varying lag time before the analysis of the sample can affect the determination of the biochemical blood parameters.^61^ Nonetheless, such studies are of particular importance when PAI is performed in a clinical environment, as globin gene mutations in humans are common, affecting around 7% of the overall population.^62^^,^^63^ Moreover, HB concentrations have been found to vary with human ethnic group,^64^^–^^66^ which could impact the acquired results in studies of mixed populations.

In summary, our findings highlight the encouraging capacity of PAI to resolve intra- and interspecies differences in HB-related blood parameters and oxygenation dynamics *in vitro* in a sensitive and label-free manner. These results suggest promising future avenues for application of PAI for HB- and oxygenation-related research studies, strengthening the position of PAI as a powerful and versatile tool in biomedicine.

## Code, Data, and Materials Availability

All associated code and raw data for this manuscript are available in the University of Cambridge repository at: https://doi.org/10.17863/CAM.55512. All materials used in the preparation of phantoms are readily available from commercial suppliers as detailed herein and further in Ref. 39.
